# From Anna University to America and to Agriculture

**DOI:** 10.6026/97320630017029

**Published:** 2021-01-31

**Authors:** Pandjassarame Kangueane

**Affiliations:** 1 Biomedical Informatics (P) Ltd, Irulan Sandy Annex, Pondicherry 607 402, India

**Keywords:** Science, Engineering, Technology, Biotechnology

## Abstract

Anna University (AU) is an awesome alma mater for attracting the attention of the invincible through awareness from education. It is a place with a plan for preparing a palace in a person's life. It is an avenue for America through adequate cGPA and Advanced GRE (AGRE) with good TOEFL score. The views,visions, modes and models of several faculty members shaped many technocrats, teachers, entrepreneurs, journalists, editors and even farmers. Technology is engineering with science. The foundation and facilities at AU is priceless. AU created the framework for Industrial Biotechnology, a truly inter disciplinary curriculum with an optimal blend of Engineering and Science (Biology especially Agriculture and Healthcare through Organic chemistry) in 1992 almost 28 years back. The place was positioned just perfect in the world for wonders to come true. The Raman auditorium (in reverence to the Nobel Laureate Sir CV Raman) reassured rational research with reasonable respect in many minds at the ACTECH (Alagappa College of Technology) under the administration of AU. The admiration, acknowledgement and accountability for the alma mater, the AU will always remain precious.

## Industrial Biotechnology at Anna University, India

My excitement to move to Chennai (formally a British colony) from Pondicherry (formally a French colony) was high, as soon as a call letter was received from Anna University for admission in Bachelor of Technology (B.Tech) in Industrial Biotechnology. Literally, my mind was prepared to see the DNA with my own eyes in 1993. Kunthala Jayaraman (KJ) is the mother of Industrial Biotechnology Education in the world who created the syllabus for it with a perfect mix of Science, Engineering and Technology at Anna University (Figure 1 - see PDF version) [1]. She was extremely active, forceful; women of wonders, and an awesome dealmaker during post Independence India. She appointed several faculty members who returned from USA after their doctoral/postdoctoral training. The subject was truly inter-disciplinary. She appointed people from various backgrounds in science and engineering, especially from Organic chemistry to Chemical Engineering so as to create a beautiful blend for Industrial Biotechnology (Figure 2 - see PDF version). She created "corpus funds" to support faculty members. The curriculum had fluid mechanics on one side and molecular biology on the other side. Thus, Industrial Biotechnology Education was novel to the world in 1992. My enthusiasm to join the programme in 1993 was tremendous. The class of 1997 (batch 1993-1997) was a small group with 20 members (Figure 3 - see PDF version). We were interacting very well with one another discussing different aspects of Industrial Biotechnology amidst confusion, exaggeration, infatuation and love. A Bachelor of Technology (B. Tech) degree in Industrial Biotechnology was confirmed on us from the Centre for Biotechnology, Alagappa College of Technology (ACTECH), ANNA UNIVERSITY in 1997 (Figure 4 - see PDF version). Most of us went to the USA either for higher education (Master of Science) in Biology through research or for work through the Information Technology sector. Many of us settled in the USA with a happy family life. Biotechnology was new to the industries in India in the 90's and the job market was weak. However, Anna University provided the foundation to explore several aspects of Industrial Biotechnology from 1993 to 1998 in our life.

## Research on Cry toxin from Bacillus thuriengis at Anna University

We (P. Kangueane, G. Kalaiselvi and R. Sachidanandam) were trying to estimate the individual population dynamics of B.t.a (Bacillus thuriengis subspeciesaizawai) and B.t.k (Bacillus thuriengis subspecies kurstaki) in a coculture system to develop a combined formulation for better pesticide activity. Similar morphology between B.t.a and B.t.k gave us hard time, determining the individual species dynamics in a co-culture system. Nonetheless, G Kalaiselvi and R Sachidanandam managed to develop a bioprocess system for the maintenance of Bacillus sphaericus 1593M in Bioreactors [3]. Kalaiselvi was cool, calm and courageous to create literature in Biotechnology during her early 20's. This is not easy.

## Research on lipase at Anna University

It was a pleasure to interact with P Gautam and BS Lakshmi on several aspects of lipase engineering. We (BS Lakshmi, P Kangueane, B Abraham and P Gautam) optimized lipase production using Candida rugosa with vegetable oils (coconut, sesame, castor, palm and sunflower) as substrates in 1999 [4]. Seasame increased lipase secretion by Candida rugosa. The lipase enzyme was known for its stereo-specificity. We (BS Lakshmi, P.Kangueane, Y Gao, YZ. Chen and P Gautam) showed the stereo-specificity of S(+) ibuprofen to Candida rugosa lipase in 2000 [5]. We (BS Lakshmi, P Kangueane, M Krishnan) also developed a simple, fast, sensitive assay method for Candida rugosa lipase using a bi-phasic reaction system [6]. We (J James, BS Lakshmi, P Gautam and P Kangueane) further showed the flap movement in different pH conditions using molecular dynamics simulation [7]. It was fun to feel the joy for working on lipase engineering with BS Lakshmi and P Gautam. P Gautam views everything with a vision for everyone. BS Lakshmi is a scientist with substance and smile.

## Bioinformatics at the National University of Singapore, Singapore (NUS)

National University of Singapore [2] was my destination in 1998 to pursue a doctoral degree in Bioinformatics (Figure 6 - see PDF version) with specific interest in MHC Informatics for the development of Vaccine Science and Technology. Tan Tinwee (Figure 5 - see PDF version) was the founding Director for the Bioinformatics Centre, NUS. His passion for Molecular Biology, Internet, networking, GRID technology and international multi-lingual Domain Name Server (iDNS) was exceptional. He was the architect of Bioinformatics in Singapore with the help of S Subbiah. The role played by Limsoon Wong, Betty Cheng and Prasanna R Kolatkar (PK) is highly remarkable. S Subbiah solved side chain packing for homology modelling with the Nobel Laureate Micheal Levitt at the Stanford University in the early 90s. He also cracked multiple sequence alignment with Steve Harrison at Harvard University in the late 80s. These methods are magical for creating miracles in modern molecular medicine. Prasanna R Kolatkar (PK) was a pleasant protein crystallographer. PK is an alumnus of the University of Texas, Austin and he worked with Micheal Rossmann (the Rossmann protein fold) at Purdue University, USA. Limsoon Wong was the master in biological data mining using Standard Mark-up Language (SML).

## Research on MHC Informatics for Short peptide vaccine design at NUS

Short antigen peptides capable of binding to host HLA molecules (Figure 7 - see PDF version) can be used to design peptide vaccines by exploiting the T-cell immunity. The design of such a cocktail vaccine is often linked to the antigen peptide diversity from viral/bacterial proteome and the host HLA allele polymorphism [8-24]. We (P Kangueane, MK Sakharkar, EC Ren and PR Kolatkar) developed a method to predict peptides binding to HLA molecules using side chain packing molecular modeling techniques developed by S. Subbiah (Kangueane *et al.* 2000). It should be noted that S Subbiah made several generous contributions towards this study. We (EC Ren, P Kangueane and PR Kolatkar) also studied the binding of mHag (minor histo-compatibility antigen involved in graft versus host disease) peptides to HLA A alleles (Ren *et al.* 2000). Betty Cheng gave her ORIGIN SGI machine to perform the modeling calculation. Her generosity is generally gentle. We (P Kangueane, MK Sakharkar, EC. Ren and P.R.Kolatkar) studied the structural principles of HLA-peptide binding using a dataset of HLA-peptide crystal structures (Kangueane *et al.* 2001). Adrian Png helped to study the type of inter-atomic interactions at the interfaces of HLA-peptide structures using a dataset (Adrian *et al.* 2002). We did develop the MIDB (MHC-peptide interaction database) to provide gleaned information (Govindarajan *et al.* 2003). Bing Zhao helped to develop a method to compress functional diversity among HLA alleles (Zhao et al. 2003a) and this was fundamental for creating a novel model to predict HLA-peptide binding (Zhao *et al.* 2003b). This later helped us to subtype HLA super-types (functional overlap among alleles) in 2005 (Kangueane *et al.* 2005). We then demonstrated the utility of this technology in the design of a gp120 peptide vaccine cocktail vaccine for NeuroAIDS in a book chapter edited by Karl Goodkin (Kangueane *et al.* 2008). Mohana Priya completed class 2 HLA-peptide binding prediction using structural principles (Mohanapriya, 2009; 2010).

## Research on Protein-protein interaction (PPI) at IISc, NTU, VITU and AIMST

An opportunity to work on the principles of protein-protein interaction (PPI) (Figure 8 - see PDF version) at the labs of P Balaram and C Ramakrishnan using a dataset of protein structural complexes during the summer of 1995 was bliss. The work with K Gunasekaran to understand principles of PPI at the Molecular Biophysics Unit, Indian Institute of Science was inspiring. Graduate students at the NANYANG Technological University, Li Lei and Cui Zhanhua showed interest to study protein-protein interaction during 2000 and 2006. Li Lei helped to explore LIGAND effect at the homo-dimer interface (Li *et al.* 2005) and homo-dimer folding (Li *et al.* 2005). Cui Zhanhua helped to identify critical interaction parameters at the heterodimer interface (Zhanhua *et al.* 2005) and differentiated hetero-dimer from homo-dimer interfaces (Zhanhua *et al.* 2005). Sajitha Lulu, a graduate student at VITU, India looked at the principles of homo-dimer folding and binding using structural data (Lulu *et al.* 2009; 2007-2008). The contribution from V Karthikraja (2009); A Suresh (2009-2010); G Sowmya (2009-2010), G Shamini (2009-2010) and Christina Nilofer (2017) in the understanding of PPI [25-38] is admirable.

## Intron research at NUS and NTU

Meena Kishore Sakharkar collaborated with Sandro J De Souza (currently in Brazil) on intron evolution right from her days at the lab of the noble laureate Walter Gilbert. It was a great pleasure to work with Meena Kishore Sakharkar during 1998-2000 on the development of IEKB (Intron-Exon knowledge base) using ExInt and GenBank (Sakharkar *et al.* 2000). We continued to work on SEGE (single exon genes in eukaryotes) during 2001-2002 (Sakharkar *et al.* 2002). Later, we developed Genome SEGE and examined their features (Sakharkar *et al.* 2004a; Sakharkar *et al.* 2004b;Sakharkar *et al.* 2005). Nidhi Dhandona, Iti Chadurvedi and Kingshuk Gosh made contributions among many others towards these developments. Lee Pern Chern, Xue Hao and Bhagavati Perumal did look into many other issues of introns in eukaryotes. This let to the continued maintenance of ExInt (Sakharkar *et al.* 2005d), development of human alternative splicing database (Sakharkar *et al.* 2004), a study on introns in tubulins (Bhagavati *et al.* 2005) and gene patterns by exon-intron combinations in the human genome (Sakharkar *et al.* 2005a). We did find that the total length in introns and intergenic DNA on each chromosome is significantly correlated to the chromosome size in both human and mouse genomes (Sakharkar *et al.* 2004; Sakharkar *et al.* 2005). These findings have implications for chromosome design and evolution in eukaryotes. We also further developed the Ugenome database containing information on exon-intron-exon in unicellular eukaryotic genomes (Sakharkar *et al.* 2005). Meena Kishore Sakharkar was a true leader in science with passion for data mining using association rules with computer-aided tools [43-55].

## Research on Gene fusion at NTU:

Meena Kishore Sakharkar was fascinated by gene fusion [39-41] in evolution through the work of Manyuan Long at the University of Chicago. Her networking skill is exceptional and she collaborated with Manyuan Long since her friendship days at the labs of Walter Gilbert (Harvard University). Gene fusion is found to mimic operons and protein-protein interfaces in prokaryotes. They are also found to exhibit multiple functions and alternative splicing. We provided a comprehensive list of fusion proteins of prokaryotic origin in the human genome (Yu *et al.* 2004). We also suggested that fusion gene products and their evolution have a key role in the selection of complex multifaceted networks (Sakharkar *et al.* 2005). The evolutionary force for gene fusion is illustrated using molecular dynamics simulation of IGPS (Yiting *et al.* 2006).

##  Service to the society, research, teaching, farming and business:

Serving as a Bioinformatics Technology Scientist (Cancer Gene Discovery),Chiron Corporation (in deputation from S*BIO Pte Ltd, Singapore),Emeryville, Bay Area, California, USA during 2001 was sensible. S*BIO Pte Ltd, Singapore is an (Economic Development Board) EDB funded company founded by Lily Chan, Singapore. S*BIO Pte Ltd acquired technology from Chiron Corporation on its cancer technology platform. This concept was enriching to the minds of many in Singapore. Discussions on this subject with Jaime Escabedo, Chief Scientific Officer (Biology) at Chiron Corporation, Emeryville, California,USA in 2001 during a period when "The Human Genome Project" was completed remains unforgettable (Figure 9 - see PDF version). There was a heated negotiation to purchase the genome data from Celera Genomics just weeks before the NIH public data was released for free. Celera went broke promptly. Thus, the private to public pressure is pleasant with pain in pleasing the people of this planet. Subsequently, my service at NANYANG Technological University as Assistant Professor (2002-2006) both teaching and developing cutting edge technologies in the field of Bioinformatics were productive. We founded Biomedical Informatics (P) Ltd in 2001. We made considerable progress providing service in the filed. Responsibility as a Professor (Visiting) at the Vellore Institute of Technology University, India (2007-2009) and Professor (Asian Institute for Medical Science and Technology, Malaysia (2009-2011) was engaging. Teaching students was fun at the organizations such as NTU, VIT and AIMST (Table 1 - see PDF version). 

Collaborations with Paul Shapshak (University of Miami, Florida) on several data mining aspects of HIV-1/AIDS related research was always filled with positive vibrations [56-59]. My association as Associate Editor,BMC Bioinformatics (a UK based Biomed Central publication since 2005) was judicious. Serving as advisors to several students leading to the award of the PhD degree by Research in the field is often sensitive [60-62]. My research contributions in Biotechnology and Bioinformatics include that of lipase engineering, vaccine science, genome design, protein-protein interactions and interfaces cholera toxins is a lifetime experience with enthusiasm. These find application in drug discovery and vaccine developments of industrial utility for social benefits. I managed to author several books (Figure 11 - see PDF version ) published by Springer, USA (2008; 2009; 2018);NOVA USA (2011) and LAP, Germany (2011) [63-69]. My excitement for farming several crops including sugarcane, peanut, black gram, coconut,lemon, rice and chrysanthemum (Chamomile) since 1990 is often special (Figure 10 - see PDF version).

## Open access to literature:

Access to available literature for advancement through the application of science for the society is secretly sensitive. The quote from BOAI "the promise was that removing access barriers would allow the world to "accelerate research, enrich education, share the learning of the rich with the poor and the poor with the rich... and lay the foundation for uniting humanity in a common intellectual conversation and quest for knowledge" explains everything. Thus, the formation of Bioinformation (Figure 12 - see PDF version), an open access (free to read) journal in Biology is engaging, entertaining and enterprising [42].

## Conclusion

This journey as a scientist, an author of scholarly materials, teacher of higher education, professor, educationalist, editor, journalist, entrepreneur, philanthropist within "possible limits", social reformer, and a farmer over the last 3 decades has been wonderful. The constant hope to bring smile in the face of the under privileged has been both challenging and awesome.
